# Central Nervous System Effects of COVID-19 in People with HIV Infection

**DOI:** 10.1007/s11904-021-00582-x

**Published:** 2021-11-29

**Authors:** Michael J. Peluso, Joanna Hellmuth, Felicia C. Chow

**Affiliations:** 1 Division of HIV, Infectious Diseases, and Global Medicine, University of California, San Francisco, CA USA; 2 Memory and Aging Center, Weill Institute for Neurosciences, Department of Neurology, University of California, San Francisco, CA USA; 3 Weill Institute for Neurosciences, Departments of Neurology and Medicine (Infectious Diseases), University of California, San Francisco, CA USA; 4 San Francisco General Hospital, 1001 Potrero Avenue, Building 1, Suite 101, CA San Francisco, USA

**Keywords:** HIV, SARS-CoV-2, COVID-19, Central nervous system

## Abstract

The convergence of the HIV and SARS-CoV-2 pandemics is an emerging field of interest. In this review, we outline the central nervous system (CNS) effects of COVID-19 in the general population and how these effects may manifest in people with HIV (PWH). We discuss the hypothetical mechanisms through which SARS-CoV-2 could impact the CNS during both the acute and recovery phases of infection and the potential selective vulnerability of PWH to these effects as a result of epidemiologic, clinical, and biologic factors. Finally, we define key research questions and considerations for the investigation of CNS sequelae of COVID-19 in PWH.

As the severe acute respiratory syndrome-coronavirus-2 (SARS-CoV-2) pandemic continues, millions of individuals worldwide remain at risk for coronavirus disease 2019 (COVID-19). The convergence between the HIV and SARS-CoV-2 pandemics is an emerging field of interest [1••, 2, 3]. Attention is needed on the effects of this novel infection on the large global population of people with HIV (PWH) — nearly 38 million people worldwide [4], many of whom will remain vulnerable to infection with SARS-CoV-2 for the foreseeable future.

HIV infection, even when well-controlled with antiretroviral therapy (ART), has long-term implications on health through mechanisms related to viral persistence, chronic inflammation, and incomplete immune reconstitution [5–11]. The central nervous system (CNS) is particularly vulnerable, due to the effects of HIV both systemically and within the CNS [9, 12–16]. This vulnerability may increase the neurologic effects of COVID-19 among PWH. Growing evidence indicates that the CNS can be impacted by SARS-CoV-2 infection, during both the acute and recovery phase of COVID-19 [17, 18 , 19•, 20–23]. However, it is currently unknown if PWH are selectively vulnerable to the CNS effects of SARS-CoV-2 infection, and if so, whether certain interventions (e.g., vaccination, COVID-19 targeted therapies) can mitigate them. In this article, we review the potential CNS effects of COVID-19 in PWH with a focus on proposed mechanisms, key research questions, and considerations for how to approach their investigation.

## Acute CNS Complications of COVID-19 in the General Population

A wide range of neurologic disorders has been observed in acute SARS-CoV-2 infection. However, most studies have focused on hospitalized patients, and less is known about the prevalence and spectrum of neurologic symptoms in COVID-19 patients who do not require hospitalization. In a UK surveillance study of 125 case notifications for neurologic and psychiatric complications among hospitalized COVID-19 patients over a 3-week period, altered mental status and cerebrovascular disease constituted the majority of syndromes reported [24]. Neurologic involvement during acute SARS-CoV-2 infection may be more common in older individuals or in those with severe illness [25, 26•]. In one study of 58 critically ill patients in the intensive care unit (ICU) in France, agitation and/or confusion were present in 69% when sedation was lifted [27]. One-third of patients had evidence of ongoing altered mental status at the time of discharge. The presence of neurologic manifestations in acute SARS-CoV-2 infection may also predict higher in-hospital mortality independent of age and disease severity [28].

It may be challenging to differentiate encephalopathy due to the direct or indirect effects of SARS-CoV-2 infection from the impact of critical illness, prolonged ICU hospitalization, and other medical issues (e.g., metabolic derangements, medication toxicity) on the brain. While some degree of encephalopathy is prevalent in hospitalized patients with COVID-19, encephalitis, defined as inflammation of the brain parenchyma by neuroimaging or cerebrospinal fluid [CSF] analysis, occurs infrequently [29–31]. In brain autopsy studies, mild non-specific inflammation (e.g., perivascular, parenchymal, and leptomeningeal lymphocytic infiltrates), acute hypoxic-ischemic injury, and microvascular changes are commonly reported, whereas frank vasculitis or meningoencephalitis are not [32, 33•, 34].

Mounting evidence points to elevated cerebrovascular risk during acute SARS-CoV-2 infection. In a study of 1916 patients who were hospitalized or sought emergency care at a single center in New York City over 2 months, 31 (1.6%) had an acute ischemic stroke compared with 3 of 1486 influenza patients (0.2%; derived from historical data) [35]. Similar to other neurologic complications of COVID-19, patients who develop cerebrovascular disease may be older and more likely to suffer from cardiometabolic comorbidities (e.g., hypertension, diabetes mellitus) and from more severe SARS CoV-2 infection [36]. In contrast, other studies have raised the specter of an association between asymptomatic or mild SARS-CoV-2 infection and elevated stroke risk in the young [37, 38].

## Neurologic Post-acute Sequelae of COVID-19 (PASC) in the General Population

Following acute illness, an unknown but likely substantial number of people experience post-acute sequelae of SARS-CoV-2 infection (PASC) [17]. Initially identified in convenience samples [18, 19•, 39–41], efforts have been made to estimate the prevalence of PASC in more representative samples, although these have typically involved short follow-up [42, 43] or have been limited to hospitalized patients [44, 45••]. Recently, preliminary data on the frequency of PASC have emerged in larger and more representative cohorts [46, 47], although true population-level studies are urgently needed. Post-acute neurologic sequelae include cognitive changes, sleep disorders, headache, neuropathy, tinnitus, vertigo, dysgeusia, anosmia/phantosmia, and dysautonomia; mood symptoms are also common [18, 39–41, 46–48].

Persistent cognitive changes are a predominant feature of CNS PASC. People can experience new difficulties staying focused (attention), holding onto and manipulating information (working memory), remembering recent events that improves with cues (executive function), and slowed thinking (processing speed). Collectively, these symptoms suggest fronto-executive brain network dysfunction. Cognitive difficulties now reported in several cohorts [39–41], including in non-hospitalized individuals [18, 19•], can persist longer than 8 months [39]. Importantly, there appears to be a considerable impact on quality of life [19•, 39], which corroborates patient-driven descriptions of PASC [49]. A clear underlying mechanism or therapeutic approach has yet to be identified.

## Neurologic Effects of COVID-19 in People with HIV

While there was initial concern that PWH would exhibit increased vulnerability to SARS-CoV-2 infection based on greater susceptibility to other respiratory pathogens [2, 50], studies to date have found comparable or lower incidence of COVID-19 among PWH [3, 51–54]. Although early studies suggested that PWH were at similar risk for severe COVID-19 as those without HIV infection [55–58], further efforts have indicated that those with a higher burden of medical comorbidities and/or incomplete immune reconstitution are likely to be at increased risk for more severe outcomes [59, 60]. No data are currently available on the clinical manifestations of the post-acute phase of SARS-CoV-2 infection and COVID-19 recovery in PWH, although studies are underway [61].

### Potential Mechanisms

**Fig. 1:**
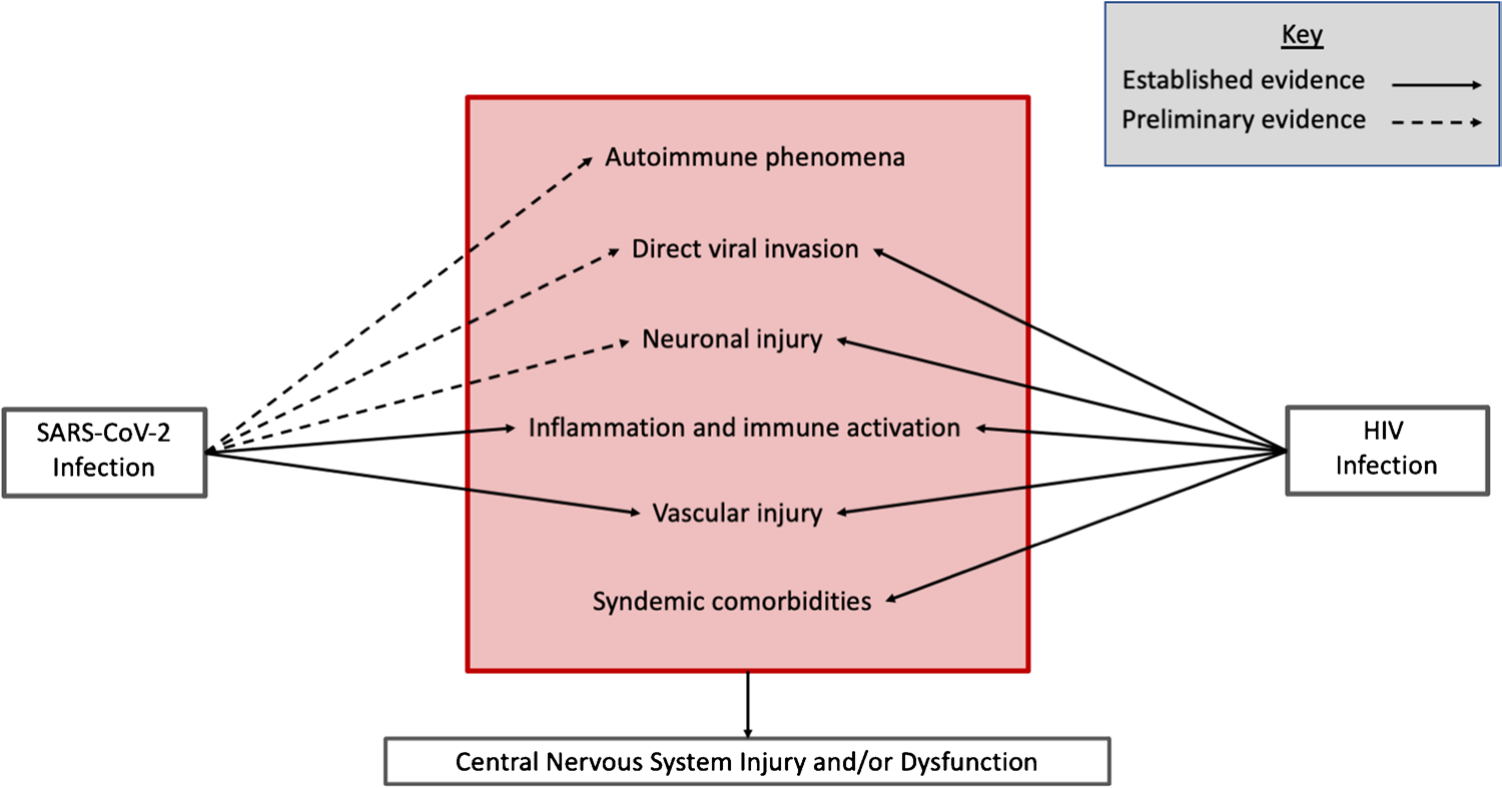
Summary of the potential overlap between known or suspected mechanisms underlying central nervous system manifestations of SARS-CoV-2 and HIV infection

Overlap or synergy in potential mechanisms driving neurologic complications of COVID-19 and HIV may place PWH with COVID-19 at higher risk for CNS dysfunction and/or injury (Fig. 1). Of particular interest is how cognitive changes associated with COVID-19 may be similar to and interface with the virally mediated cognitive disorder of HIV, known as HIV-associated neurocognitive disorder (HAND). The clinical presentation of HAND, which may impact up to one-third of PWH on ART [62], is strikingly similar to the executive dysfunction seen following COVID-19 [63]. However, one notable difference between patients with COVID-19 and HIV-associated cognitive impairment is the temporal onset of symptoms. Based on our current knowledge, cognitive changes appear acutely or within the first few months following COVID-19, while HAND can develop after years of sustained viral suppression on antiretroviral therapy. This may hint at a critical distinction between the impact of these two viruses on the CNS. HIV causes a chronic infection and persists in latently infected cells in reservoirs established early in infection, including in the CNS. The presence of persistent HIV-infected cells in CSF has been linked to worse cognition in PWH [64]. In contrast, while we are still early in our understanding of the trajectory of SARS-CoV-2 infection and the potential for viral persistence [65], including in immunocompromised patients [66, 67] and patients with severe disease [68], most patients appear to be able to effectively clear SARS-CoV-2 infection. However, if SARS-CoV-2 can persist in the CNS long term, as has been shown for other human coronaviruses [69, 70], the prospect of ongoing neurologic injury and delayed neurologic dysfunction may be greater. Furthermore, mechanisms underlying encephalopathy in the setting of acute SARS-CoV-2 infection may diminish existing cognitive reserves and unmask or magnify cognitive impairment in PWH. In our own clinical practice, some patients with HAND have reported worsening of cognitive symptoms following COVID-19.

Preliminary data and expert opinion suggest that one or more of several mechanisms might contribute to the potential neurologic effects of COVID-19 in PWH:

1. *Altered immune response, inflammation, and immune activation:* Systemic and CNS inflammation and immune activation, which can remain elevated in PWH even after viral suppression with ART [71–74], have been implicated in the pathogenesis of complications associated with long-term HIV infection, including HAND [75–77]. Activation of inflammatory pathways in acute SARS-CoV-2 infection could compound the impact of chronic inflammation on the CNS in PWH [78–80]. One study demonstrated marked intrathecal inflammation, measured by CSF neopterin and b_2_-microglobulin, in hospitalized patients with COVID-19 and neurologic symptoms, although CSF pleocytosis, elevated IgG index, and evidence of blood–brain barrier disruption were absent [81]. Furthermore, immune activation and inflammation, either systemically or in the tissues, may persist after acute COVID-19 [17, 39]. Levels of soluble markers of inflammation (e.g., interleukin—10 (IL-10), macrophage-inflammatory protein-1, interferon-inducible protein -10 (IP-10)) have been shown to be elevated in SARS-CoV-2 convalescent plasma donors compared with healthy blood donors without COVID-19 [82•]. If this is the case, ongoing immune dysregulation may contribute to post-COVID cognitive changes, to which PWH might be particularly susceptible.
2. *Direct viral invasion and neuronal injury:* Post-mortem and human brain organoid studies have demonstrated direct SARS-CoV-2 invasion into the CNS with resulting neuronal death [33•, 83•, 84]. In one postmortem study, SARS-CoV-2 infection was identified in areas of cerebral microinfarcts, raising the possibility that direct viral invasion may contribute to CNS injury and neurologic symptoms in COVID-19 [83•]. However, despite evidence of neuroinvasive potential of SARS-CoV-2, we do not yet know the frequency of this and the precise relationship between neuroinvasion and the development of acute and post-acute neurologic sequelae.
  Whereas identification of SARS-CoV-2 RNA from CSF has been rare [30, 81, 84, 85•], HIV RNA is readily measured in the CSF within days to weeks of infection [15, 86] and in persons with untreated chronic infection [87]. The degree of CNS injury related to direct viral invasion, immune activation, and neuroinflammation may be dictated by events during acute HIV infection, similar to the establishment of an immune set point early in HIV infection, which predicts later decline and disease progression. Very early ART initiation may mitigate HIV-associated CNS injury [88, 89], although this is often not logistically feasible, and risk for HAND persists even with sustained viral suppression [90].
  The presence and severity of HAND is associated with markers of brain injury, including neurofilament light chain (NFL), a protein present in myelinated axons of neurons [91, 92]. Similarly, in a study of hospitalized patients with moderate to severe COVID-19, NFL and/or glial fibrillary acidic protein (GFAP), an astroglial injury/activation marker, were elevated [93•]. This finding implies that CNS injury, rather than dysfunction of intact brain cells, occurs in some patients with moderate-to-severe COVID-19. The clinical consequences of this injury, which may be related to direct viral invasion or other mechanisms (e.g., persistent immune activation), could be especially relevant to PWH with ongoing HIV-related neurologic injury.
3. *Post-infectious autoimmunity:* Given the infrequent detection of SARS-CoV-2 neuroinvasion, some data suggest that CNS injury or dysfunction is caused by immune-mediated mechanisms that occur secondary to systemic infection. A recent study found that 5 of 7 hospitalized COVID-19 patients with a spectrum of neurologic issues displayed evidence of CNS autoantibodies [23]. Further work is needed to ascertain whether autoimmunity plays a role in PASC. Autoimmune phenomena are sometimes associated with the immune dysregulation that characterizes HIV infection [93•, 94–96], and it is unknown whether the autoimmune risk in SARS-CoV-2 is modified by HIV infection.
4. *Neurovascular injury:* The long-term projections for brain health and recovery after SARS-CoV-2 infection may be influenced by mechanisms driving neurovascular risk during acute infection, including a dysfunctional immunologic and endothelial response triggering cerebral microvascular injury and thrombosis [34]. Furthermore, subclinical microvascular injury triggered by acute infection may not become clinically apparent until later in the course of recovery. As has been shown with other systemic infections [97, 98], cerebrovascular risk associated with COVID-19 may also remain elevated beyond the acute period.
  The proposed mechanisms by which SARS-CoV-2 may lead to elevated cerebrovascular risk may be particularly relevant for PWH. PWH contend with a heightened risk of cerebrovascular injury, attributed in part to chronic systemic immune activation and inflammation [7, 9]. Cerebrovascular disease and associated cardiovascular comorbidities, in turn, are major contributors to HIV-associated cognitive impairment. In fact, cardiovascular risk factors may play a greater role in the development of cognitive impairment than HIV disease activity [99, 100], especially among individuals with treated infection. Thus, for PWH, the immunologic and inflammatory response to COVID-19 may serve as an unwelcome “second hit,” further compounding cerebrovascular risk and cognitive injury in this at-risk population. Studies have supported the concept of a cumulative infectious burden contributing to cerebrovascular risk, in which individual infections may not surpass thresholds to impact stroke risk but several infections in aggregate can [101].
5. *Syndemic comorbidities:* Syndemic comorbidities in PWH, including substance use and metabolic disorders, may increase risk for contracting SARS-CoV-2 infection and lead to more severe disease and worse clinical outcomes [1••, 102]. These comorbidities may also contribute to the development of CNS injury in PWH with SARS-CoV-2 infection. Use of some substances has been linked to structural brain injury and long-term impairments in cognitive function in PWH [103, 104], even after initiation of ART [105]. PWH who use stimulants can experience challenges navigating the HIV care continuum, resulting in barriers to achieving and maintaining virologic suppression [106], which is critical to mitigating the inflammatory response in HIV. Furthermore, plasma and CSF inflammation and immune activation are heightened among PWH with a history of current and prior substance use [105, 107]. Amplification of immune dysregulation is one plausible mechanism by which substance use could increase susceptibility to CNS injury in PWH. Similarly, altered immune responses and chronic inflammation observed in metabolic disorders, including obesity and type 2 diabetes mellitus [108–110], are proposed contributing factors to increased risk of SARS-CoV-2 infection and of severe disease. As with substance use, these pathways, which may mediate the association between metabolic disorders and HAND [111], could also intensify CNS injury associated with COVID-19 in PWH.

## Key Considerations for Research

Understanding the epidemiology and pathogenesis of neurologic complications of SARS CoV-2 infection will inform our approach to investigating potential CNS effects of COVID-19 in PWH. The optimal study approach will depend on the specific research questions that are of greatest interest to PWH and their providers. Although there are many potential questions related to CNS effects of COVID-19 in PWH (Table 1), we believe four are key:

1. Are PWH *more vulnerable* to CNS effects of SARS-CoV-2 infection than the general population?
2. Are CNS manifestations of COVID-19 during the acute or recovery phase *more severe or more persistent* in PWH compared to people without HIV?
3. Are certain pathogenic mechanisms that underlie the CNS effects of SARS-CoV-2 infection *potentiated* in PWH?
4. Which interventions, including SARS-CoV-2 preventative or therapeutic interventions or HIV-targeting interventions, could *mitigate* the impact of neurologic complications of SARS-CoV-2 infection in PWH?

**Table 1:** Key research questions regarding CNS manifestations of SARS-CoV-2 infection in people with HIV infection

Epidemiologic questions
What is the case definition for CNS manifestations of SARS-CoV-2 infection?
What is the prevalence of CNS manifestations of SARS-CoV-2 infection in people with HIV?
Are CNS manifestations of SARS-CoV-2 infection more common, severe, and/or persistent in PWH compared with people without HIV?
What are the determinants of the presence, severity, and duration of CNS manifestations of SARS-CoV-2 infection in PWH? Do these differ from the determinants in people without HIV infection?
What are the predictors of the presence, severity, and duration of CNS manifestations of SARS-CoV-2 infection in PWH? Do these differ from the predictors in people without HIV infection?
Biologic questions
What biologic mechanisms underlie the CNS effects of SARS-CoV-2 infection?
Are certain pathogenic mechanisms that underlie the CNS effects of SARS-CoV-2 infection potentiated in PWH?
Are there unique pathogenic mechanisms that contribute to CNS effects of SARS-CoV-2 infection in PWH?
Does HIV virologic (i.e., suppressed or unsuppressed) and immunologic status (i.e., proximal or nadir CD4 + T cell count) affect the development of CNS effects of SARS-CoV-2 infection)?
Clinical science questions
Does SARS-CoV-2 vaccination or acute treatment mitigate the development of neurologic complications during the acute or post-acute phases of infection?
What interventions will be effective in managing and treating post-acute neurologic complications of SARS-CoV-2 infection in PWH? Are any interventions likely to uniquely benefit PWH?

### Representative Sampling and Meaningful Comparator Groups

While descriptive analyses can characterize acute and post-acute neurologic COVID-19 complications in PWH to inform further research efforts, calculating an accurate prevalence estimate of CNS complications will require large, representative cohorts. Such cohorts should be agnostic to the presence of neurologic symptoms at the time of enrollment to avoid selection bias. To date, many descriptions of COVID-19 and its sequelae have utilized case series or convenience samples, which may not capture the true frequency of neurologic manifestations. Similarly, a prevalence estimate of neurologic complications of COVID-19 within the population of PWH in a certain geographic region will require cohorts representative of the full spectrum of individuals with HIV infection who acquire SARS-CoV-2.

Equally important to the epidemiologic questions is the utilization of meaningful comparator groups. An ideal study design would include clinical and biologic data from prior to and following the SARS-CoV-2 pandemic. Large observational cohorts of HIV infection [112, 113] that include a subset of individuals who developed COVID-19 could be leveraged to conduct such studies. Studies that are unable to evaluate pre-COVID-19 timepoints in PWH will require thoughtfully selected comparator groups. Depending on the question for investigation, this might be people with COVID-19 without HIV infection, PWH without COVID-19, or people with neither a history of HIV nor SARS-CoV-2 infection. Substantial effort will be required to ensure that these comparator groups are constructed in a way that minimizes the risk of selection biases that could arise from differential forces driving recruitment and inclusion. In some settings, oversampling of certain groups may be needed to facilitate inclusion of comparator groups that are sociodemographically similar to those with HIV infection, especially in light of the changing HIV epidemic. In the USA, this might include purposeful oversampling of specific demographic groups (e.g., Black and Latinx individuals, women) or groups with certain risk factors (e.g., men who have sex with men, injection drug users). In addition, as the neurologic burden of HIV infection has been increasingly recognized in resource-limited settings, global populations must be included in these research efforts.

### Data Sources and Quality of Measurements

While electronic medical record (EMR)-derived data can be used to measure neurologic effects of COVID-19 among those hospitalized during the acute period, variability in access to care, provider assessments, symptom screening, and participant reporting will complicate the use of these data for non-hospitalized individuals and for all individuals during the post-acute period. In addition, the quality of measurements is relevant. Most existing studies have queried participant symptoms through self-report or interview. Few have included objective testing, which will require a combination of structured assessments, neurologic examination, and neuropsychological testing. In our experience, routine cognitive screening tests developed for the detection of dementia, such as the Montreal Cognitive Assessment and Mini-Mental State Examination, may not utilize sensitive enough thresholds to detect the cognitive inefficiencies observed following COVID-19, particularly as this population includes a younger age range of patients [17]. Collection of data on the abovementioned syndemic comorbidities, among other conditions that may co-occur with HIV infection (e.g., cardiovascular disease, psychiatric illness) and can affect brain health, will be pertinent to evaluating the independent contributions of COVID-19 to CNS injury and whether HIV modifies this relationship.

In line with the importance of studying population-based cohorts that reflect the distribution of demographic and socioeconomic factors in communities affected by COVID-19, these considerations are also germane to the investigation of potential CNS effects of COVID-19. Some of these factors have been associated not only with risk of SARS-CoV-2 infection but also with differential outcomes during both the acute and recovery phases of COVID-19 [47, 114–116] and with the development or severity of HAND [117–119]. Selection of neuropsychological tests that can measure cognitive function across diverse populations will be critical in the assessment of cognitive effects of SARS-CoV-2 infection [120]. Furthermore, data on social determinants of brain health (e.g., nativity and acculturation; language; education; literacy; psychosocial stress; occupation; economic and financial status; residential characteristics) will be essential to accurately interpret neuropsychological test results [120]. Considerations of these factors and how they impact brain health have been pioneered for decades in HIV research [117, 121–123]. Drawing from lessons learned in international multi-site studies [124, 125] on the evaluation of cognitive function in PWH across countries, languages, and educational backgrounds will also inform the approach to cognitive testing in patients with COVID-19.

### Biologic and Advanced Clinical Measurements

To understand the mechanisms driving neurologic involvement of SARS CoV-2 infection and interactions in PWH, we will need longitudinal data on systemic and CNS inflammation, neuronal injury, the presence of autoantibodies, and other biologic factors beyond the acute infection period. In addition to blood measurements, CSF analyses may be critical. For example, some patients with COVID-19 and neurologic complications have displayed differential inflammatory profiles in CSF compared with plasma, suggesting compartmentalized CNS immunologic responses may contribute to neurologic disease [23]. Available neuroimaging data in COVID-19 are almost exclusively in patients with acute SARS-CoV-2 infection [126, 127]. Information on neuroimaging findings in post-acute infection is limited. One study demonstrated volumetric and microstructural differences on brain MRI in patients with COVID-19 at 3 months after infection compared with age- and sex-matched controls without COVID-19 [128]. Neuroimaging techniques that assess longitudinal brain structure, function, and cerebrovascular physiology in the post-acute period may yield insights into the pathogenesis of CNS injury in COVID-19, as they have in the field of HIV [129–133].

### Representation of PWH in Therapeutic Studies

Through intense community advocacy, PWH were included in initial SARS-CoV-2 vaccine trials [134–136], although the numbers were often small [137]. In order to understand the potential impact of SARS-CoV-2 preventative or therapeutic interventions on CNS sequelae of COVID-19 in PWH, PWH will need to be eligible for and engaged in clinical trials. Subgroup analyses should examine the effects of these interventions on PWH, who may exhibit distinct responses based on different underlying risks and comorbidities. Furthermore, studies of interventions for both symptom management and treatment that may be specific to PWH will need to be considered, designed, and implemented.

## Conclusion

The convergence of the dual HIV and SARS-CoV-2 pandemics has highlighted the urgent need for research on potential interactions between these two infections. Much of our knowledge of how viruses impact the brain and brain function derives directly from HIV research. Leveraging research infrastructure and scientific methods developed over decades of effort in the fight against HIV has allowed for rapid, hypothesis-driven study of the CNS effects of COVID-19. Studies investigating the determinants of CNS effects of SARS-CoV-2 infection in PWH and whether HIV infection potentiates the mechanisms underlying these effects will further illuminate our understanding of virally-mediated CNS disease and could potentially improve the quality of life of millions of PWH worldwide.
